# Rapid commissioning of Brainlab Elements for SRS: end-to-end evaluation of Monte Carlo and pencil beam algorithm across homogeneous and heterogeneous phantom environments

**DOI:** 10.3389/fonc.2026.1893085

**Published:** 2026-08-11

**Authors:** Bhuvaneswari Narayanan, Daniel Saenz, Nikos Papanikolaou, Neil Kirby, Doug Soltesz, Eva Galvan, Richard Tuli, Karl Rasmussen, Holly Paschal, Nema Bassiri-Gharb

**Affiliations:** Department of Radiation Oncology, University of Texas at San Antonio, San Antonio, TX, United States

**Keywords:** Brainlab, commissioning, end-to-end testing, SRS, treatment planning system

## Abstract

**Purpose:**

Brainlab released a reference beam model that accelerates Brainlab Elements treatment planning system (TPS) commissioning process for stereotactic radiosurgery (SRS) using an Elekta Versa HD linear accelerator. A series of end-to-end (E2E) phantom studies were performed to evaluate the Monte Carlo (MC) and pencil beam (PB) dose calculation algorithms using the reference beam model released by Brainlab with Elements v4.0 and v4.5.

**Methods:**

A reduced set of water tank measurements of percent depth dose (PDD), beam profiles, multileaf collimator (MLC) gap, and output factors were taken using a PTW 0.125-cc Semiflex ionization chamber, PTW Si-diode, and PTW Microdiamond chamber for field sizes including 6 × 6, 10 × 10, 30 × 30, and 100 × 100 mm^2^ using 6MV and 6MV flattening filter-free (FFF) beams. MPPG 5.b testing was performed after commissioning to validate TPS performance. A set of RTsafe cranial and spine phantoms and an Exradin A16 ionization chamber was used for E2E testing. The cranial phantom was used to evaluate single target and dynamic conformal arc therapy (DCAT) cranial planning, and spine phantom filled with water, air, and air with a steel rod insert was used to evaluate spine SRS planning. MC and PB (v4.0 and v4.5) dose calculation algorithms in Brainlab Elements were used to evaluate the planning modules across all phantom environments.

**Results:**

All commissioning validation tests satisfied the MPPG 5.b tolerance criteria. The commissioning process took a qualified medical physicist (QMP) and a medical physics resident 1 day to complete. MC dose calculation (2%/1 mm) demonstrated clinically acceptable agreement within 3% of measured dose for E2E tests. In-field PB calculations were within 5%. PB calculated plans showed some improvement with measured values when upgrading the beam model from v4.0 to v4.5.

**Conclusion:**

The reference beam model provided an efficient framework for commissioning the Brainlab Elements TPS. Brainlab Elements’ MC dose calculation algorithm provides reliable accuracy across clinically relevant heterogenous and small field conditions.

## 1 Introduction

Stereotactic radiosurgery (SRS) and stereotactic radiotherapy (SRT) deliver a high dose of radiation ranging from a single course (SRS) to five fractions of treatment (SRT). SRS and SRT treatments utilize non-coplanar couch angles, hot spots ranging up to (and exceeding) 130% of the prescribed dose, high conformality, and steep dose gradients to maximize the dose delivered to the tumor while sparing as much of the healthy tissue as practicable (1).

Commissioning a treatment planning system (TPS) for SRS traditionally requires extensive beam data acquisition and beam modeling. Typically, this process can take up to 2–4 weeks for a single photon energy (2, 3). The volume of required beam scan data is large, and beam modeling is susceptible to human error (4). Accurate measurement of small field output factors, percent depth dose (PDD), and beam profiles are challenging and crucial to SRS planning, as many SRS/SRT field sizes are less than 1 cm^2^ (5–7). Small field measurements are highly susceptible to detector selection effects, setup uncertainties, and human error. Inaccuracies in dose calculation arise from subtle mistakes in beam data acquisition. To streamline the commissioning process, several vendors have introduced beam data for comparison that allow institutions to match their linear accelerator to a standardized beam model (8–11). Brainlab has developed a reference beam model for the Elements treatment planning system (11).

Another important consideration would be the choice of dose calculation algorithm. Pencil beam (PB) dose calculation algorithms are susceptible to inaccurate beam modeling and particle transport for small fields, commonly seen in SRS planning, and scattering conditions (12). Monte Carlo (MC) algorithms are computationally more expensive and take longer to run but provide a more realistic representation of radiation transport and are considered to be the most accurate method to calculate dose in treatment planning systems (12).

The purpose of this study was to evaluate the dose calculation accuracy for Brainlab Elements (Brainlab SE, Munich, Germany) cranial SRS and spine SRS using the reference beam model for both 6MV and 6FFF energies using a series of phantoms and ion chamber measurements. We tested the accuracy of the MC and PB algorithms for small field sizes (FS), containing one minimum leaf width, and large field treatment plans, plans containing multiple targets. MC and PB calculation accuracy for targets in an inhomogeneous media, respectively, were evaluated. Furthermore, a phantom with a metal insert was created to determine the dose calculation accuracy at a bone/metal interface (replicating surgical hardware or other metal implants).

## 2 Methods and materials

There were four basic aims for this study, namely: (1) quick commissioning Brainlab Elements using a reference beam model, (2) evaluation of cranial and spinal planning in a tissue-equivalent and water-filled phantom, respectively, (3) evaluation of spinal planning in an air-filled phantom, and (4) evaluation of spinal planning in an air-filled phantom with a metal insert. The Brainlab Elements’ reference beam model was implemented by measuring a reduced number of beam profiles, output factors, and PDDs. The efficacy of the beam model was evaluated by creating a series of end-to-end (E2E) tests with plans using the RTsafe (RTsafe Athens, Greece) cranial and spinal phantoms. All phantom imaging was performed using the Siemens SOMATOM go.Open Pro (Siemens AG, Munich, Germany). Plans were created using PB (v4.0 and v4.5) and MC algorithms, and an E2E evaluation was performed using an Exradin A16 MicroPoint ionization chamber (Standard Imaging, Middleton, Wisconsin). Corrected chamber measurements were compared to the mean dose of the contoured air cavity, which represents the active volume of the chamber, in the phantom from each treatment plan. Plans were created and evaluated using a 6MV and a 6FFF beam model developed with Brainlab Elements Cranial SRS or Elements Spine SRS planning applications. All MC plans were calculated with an uncertainty of 2% and a grid size of 1 mm. PB plans were calculated using the reference beam models v4.0 and v4.5, which have improved PB modeling. The PB v4.0 plans were copied and recalculated using the PB v4.5 beam model and algorithm. The plans were delivered on an Elekta Versa HD (Elekta AB, Stockholm, Sweden) equipped with Brainlab ExacTrac Dynamic for image guidance. The basis of Brainlab Elements planning allows the user to move three categories of sliders to develop SRS plans: weighting, normal tissue sparing, and modulation. Contoured organs at risk (OARs) also have sliders that are able to affect planning. Users can move the “Guardian” slider from 0 to 100 to manipulate the optimizer and place importance on OARs within the plan (100 being the highest weighted and most important task). Each OAR constraint has three settings: off, smart, and strict. Users can decide which OARs to avoid and ignore when treatment planning. Furthermore, there is an “advanced editing” feature which allows further customization to planning around the target volume, but that feature was not used and is not within the scope of this study.

The first aim was to commission the TPS using the reference beam model provided by Brainlab. **Table 1** outlines a summary of all the beam scans with the associated detectors and setups. Three dosimeters were used to collect beam data: PTW Semiflex 0.125 cc (PTW Freiburg GmbH, Freiburg, Germany), PTW 60012 unshielded Si diode, and PTW microdiamond. All beam data were collected using a PTW BEAMSCAN water tank at 90-cm source-to-surface distance (SSD). Beam profiles, multileaf collimator (MLC) gap measurements, and outputs were all taken at 10-cm depth. The reported measurements were taken for 6MV and 6FFF beam energies. The PTW Semiflex 0.125 cc was used to collect percent depth dose (PDD) for 100 × 100 mm^2^ FS. Beam profiles of 100 × 100 mm^2^ and 200 × 200 mm^2^ and outputs for 30 × 30 mm^2^, 100 × 100 mm^2^, and 200 × 200 mm^2^ were taken. All Semiflex measurements were performed with the collimator set at 0°.

**Table 1:** Specific detectors used to take beam commissioning measurements and their associated parameters.

Detector	Measurement	Field size (mm^2^)
Semiflex 0.125 cc	Percent depth dose	100 × 100
	Beam profile	200 × 200 100 × 100
	Output factor	200 × 200 100 × 100 30 × 30
	Sweeping gap MLC	Vendor provided DICOM
PTW Si Diode and microdiamond	PDD	6 × 6 (coll = 0, 90 degrees)
	Beam profile	200 × 200 100 × 100 30 × 30 10 × 10 6 × 6 (coll = 0, 90 degrees)
	Output factor	30 × 30 10 × 10 6 × 6 (coll = 0, 90 degrees)

The solid state detectors (Si diode and Microdiamond) were used to measure small field beam outputs, profiles, and PDDs. Small field (FS < 30 × 30 mm^2^) measurements were performed at the point of maximum detector response. Both detectors were set up independently of one another to verify small field measurements. Beam profiles were taken for 200 × 200-, 100 × 100-, 30 × 30-, 10 × 10-, and 6 × 6-mm^2^ fields. PDDs were taken for a 6 × 6-mm^2^ field. Output factors were taken for 30 × 30-, 10 × 10-, and 6 × 6-mm^2^ fields. Output factors, PDDs and beam profiles were taken at an additional collimator angle of 90°for 6 × 6-mm^2^ FS. The average values of the Si Diode and Microdiamond chamber were taken and input into the TPS and used for beam modeling.

The Versa HD uses the Agility MLC, which has 160 leaves with rounded edges, each with a 5-mm projected width at isocenter. Leaves move in the X-direction. The Y-jaw moves independently and defines the Y-direction for every field size. The X and Y banks are defined by the rounded MLC and jaw, respectively, for the Versa HD (13). A positional uncertainty analysis was performed by shifting the active chamber +/- 0.5 mm from its intended position. The shifted chamber set up was compared against the dose received in its initial position. Note that 0.5 mm was the tolerance set on the BrainLab ExacTrac dynamic system.

Sweeping gap MLC measurements were taken using DICOM fields provided by Brainlab to model MLC leaf end transmission. The PB v4.0 and v4.5 software used asynchronous gap fields (ASG). Note that synchronous gap and asynchronous gap fields (SG-aSG20) can be used with PB v4.5 beam model. Fields were imported into the Elekta MOSAIQ R&V system and measured in a water tank at 90-cm SSD and 10-cm depth using the Semiflex 0.125 cc ionization chamber. The raw charge data was uploaded into Brainlab Elements to compute MLC leaf end transmission for each beam model.

**Figure 1:**
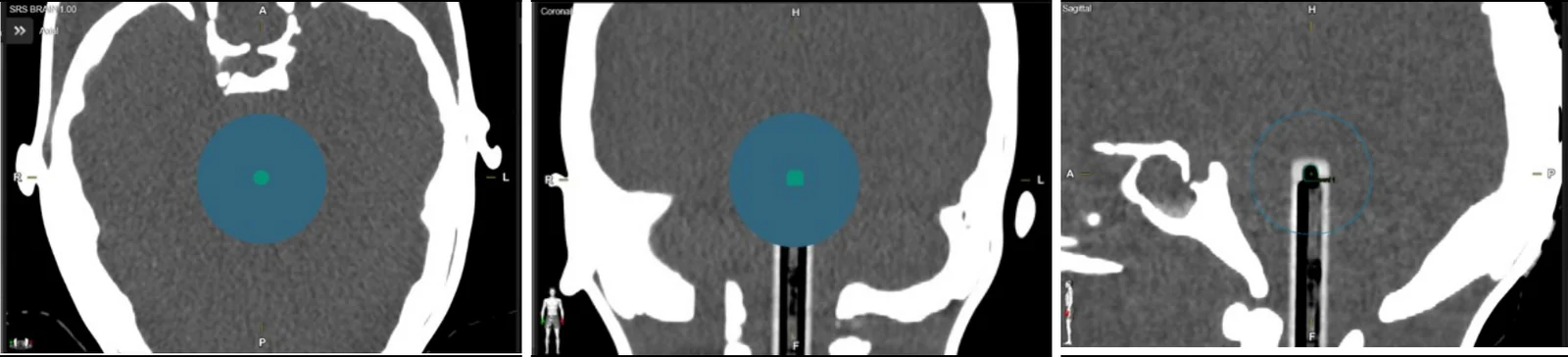
The cranial phantom with axial (Left) and coronal (Center) slices of the PTV large (blue) and PTV small (green) is displayed. A sagittal display (Right) of the A16 insert is displayed with out color-washed PTV targets.

A daisy chain method was used with the 30 × 30-mm^2^ field to relate the small field output factors measured by the solid-state detectors to the reference 100 × 100-mm^2^ field. Detector-specific small field correction factors from the IAEA TRS 483 report (13) were also used to correct the output factors. After beam modeling was performed, testing was done to ensure that TPS met the standards outlined by AAPM MPPG 5.b. As part of the MPPG 5.b testing recommendations, a variety of test plans were developed for patient-specific QA on the PTW Octavius with the 1000 SRS 2D array insert. Tests that were performed included verification of PDD ratios along central axis, off axis ratio at various depths, off axis field measurements, asymmetric field measurements, oblique incident fields, small field PDDs, small field output factors, and VMAT plans underwent patient specific QA using 3%/1 mm gamma index. Plans were created using both small and large fields with the highest possible modulation setting. Our objective for the second aim was to evaluate the efficacy of the beam model, outlined previously, in a water-filled cranial and spine phantom using both a PB and MC calculation algorithm with 6MV and 6FFF beam energies. The cranial planning process was bifurcated into two techniques: dynamic conformal arc therapy (DCAT), which was used for multiple brain metastases (multimet) planning (Multimet Plan), and single target planning. There were two types of cranial single target plans: large planning target volume (PTV) (PTV-Large volume = 32.6 cc) and small PTV (PTV-Small volume = 0.08 cc) as well as two types of DCAT multimet plans: sensitive volume in target (multimet) and sensitive volume outside target (multimet avoid). The PTV-Large and PTV-Small in the cranial phantom environment are displayed in **Figure 1**. The PTV-Small was a 1-mm expansion of the contoured active volume of the ion chamber.

In addition to the small and large PTVs, there were five cranial gross tumor volumes (GTVs) and PTVs contoured in the cranial phantom, which were solely used for DCAT planning using Elements Multiple Brain Mets SRS (v 4.0). Target location from the isocenter, located at the center of the active volume, ranged from 4.2 to 6.6 cm. The cranial GTVs and PTVs were randomly drawn around the active volume in a non-coplanar fashion. The Multimet Plan included the five cranial PTVs in addition to the PTV with the contoured active volume described previously. The Multimet Avoid plan prescribed dose to the five cranial PTVs and avoided the PTV-Large and PTV-Small. Prescriptions for all cranial plans were 20 Gy delivered in one fraction. There were five couch angles used in the optimizer for each plan. The plans were optimized with the Brainlab Elements planning sliders remaining in the default position (middle).

**Figure 2:**
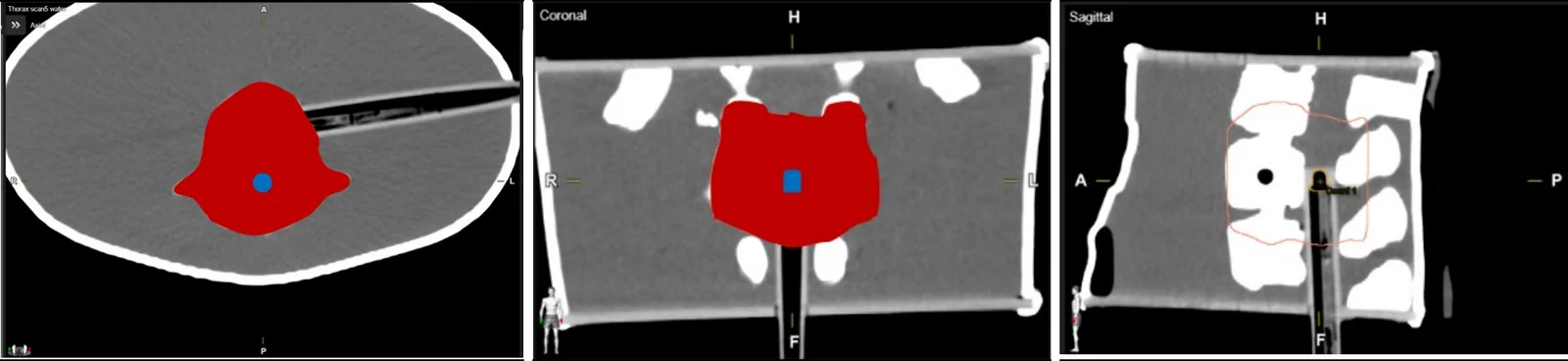
The spine phantom with axial (Left) and coronal (Center) slices of the PTV spine (red) and PTV small (blue) is displayed. A sagittal display (Right) of the A16 insert is displayed with out color-washed PTV targets. The avoid structure runs down the spinal cord region, as seen in (Right), where the chamber insert is located. Note that there are two inserts for the A16 ion chamber: one runs through the spinal canal, and the other intersects the vertebrae in the phantom.

Spinal cord SRS plans were created in a spine phantom filled with water. Three types of spine plans were generated, namely: PTV with avoid structure (PTV-Avoid), spine PTV, and simultaneous integrated boost (SIB). Targets in the spinal cord phantom were prescribed to 8 Gy in one fraction. The spinal phantom is displayed in **Figure 2**. PTV-Small (volume = 0.27 cc) was created from a 1-mm expansion of the contoured active volume of the ion chamber (volume = 0.11 cc), and PTV-Spine (volume = 40.75 cc) was a contour of a single vertebrae in the thorax phantom. The SIB plan was generated by treating the PTV-Spine to 8 Gy and PTV-Small to 10 Gy. Furthermore, PTV-Avoid (volume = 39.14 cc) was generated by subtracting PTV-Small from PTV-Spine (**Table 2**). The avoid plan was created by prescribing 8 Gy to the PTV-Avoid structure. There was a “strict” constraint of D1.6cc <4 Gy and Dmax <5 Gy, with the Guardian slider was set to 50. The Elements planning sliders were unaltered and remained in the default position.

**Table 2:** The TPS calculations using both PB and MC algorithms were based on the average dose to the active volume of the ion chamber and compared with corrected measurements taken with an A16 ion chamber in a cranial phantom.

Aim 2 cranial plans			TPS (Gy)	Measured (Gy)	Difference (%)
Cranial small	6MV	PB v4.0	23.23	24.47	-5.34
		PB v4.5	23.48		-4.22
		MC	22.46		-8.95
	6FFF	PB v4.0	22.52	21.26	5.60
		PB v4.5	22.77		6.63
		MC	21.25		-0.05
Cranial large	6MV	PB v4.0	22.14	22.26	-0.54
		PB v4.5	22.19		-0.32
		MC	22.38		0.54
	6FFF	PB v4.0	20.07	19.98	0.45
		PB v4.5	19.97		-0.05
		MC	19.97		-0.05
Multimet	6MV	PB v4.0	10.22	10.32	-0.98
		PB v4.5	10.28		-0.39
		MC	10.32		0.00
	6FFF	PB v4.0	10.06	9.89	1.69
		PB v4.5	10.01		1.20
		MC	9.97		0.80
Multimet avoid	6MV	PB v4.0	1.09	1.22	-11.93
		PB v4.5	1.14		-7.02
		MC	1.06		-15.09
	6FFF	PB v4.0	1.32	1.31	0.76
		PB v4.5	1.33		1.50
		MC	1.29		-1.55

The third aim of this study was to evaluate the efficacy of the beam model and TPS in an inhomogeneous environment. The water-filled spinal phantom, previously described and displayed in **Figure 2**, was drained to create an environment containing air–bone interfaces. Plans with three types of PTV were created, namely: PTV-Large, PTV-Avoid, and SIB. Target contours, avoid structure, planning methods, and prescription protocols were copied from the water-filled spine phantom from the second aim to the air-filled spine phantom study. The PTV-Spine target was expanded in the superior–inferior direction by half the vertebral length in each direction. The PTV-Large volume was 99.2 cc. PTV-Small was a 1-mm expansion of the active volume. The SIB plan and PTV-Avoid plan were constructed in the same manner as described from aim 2. The only beam model evaluated for this section was 6FFF.

The fourth aim was to evaluate the performance of the beam model and TPS in an environment containing a metal insert. A steel rod was inserted into the air-filled spine phantom. To model the electron density (ED) in the TPS, the steel rod was contoured and assigned an ED of 5.00, within the “Tissue Modeling” module in Brainlab Elements. There were three PTVs generated in the metal spine phantom environment, namely: PTV-Spine-Metal (volume = 49.57 cc), PTV-Small-Metal (volume = 0.27 cc), and PTV-Large-Metal (volume = 255.79 cc).

**Figure 3:**
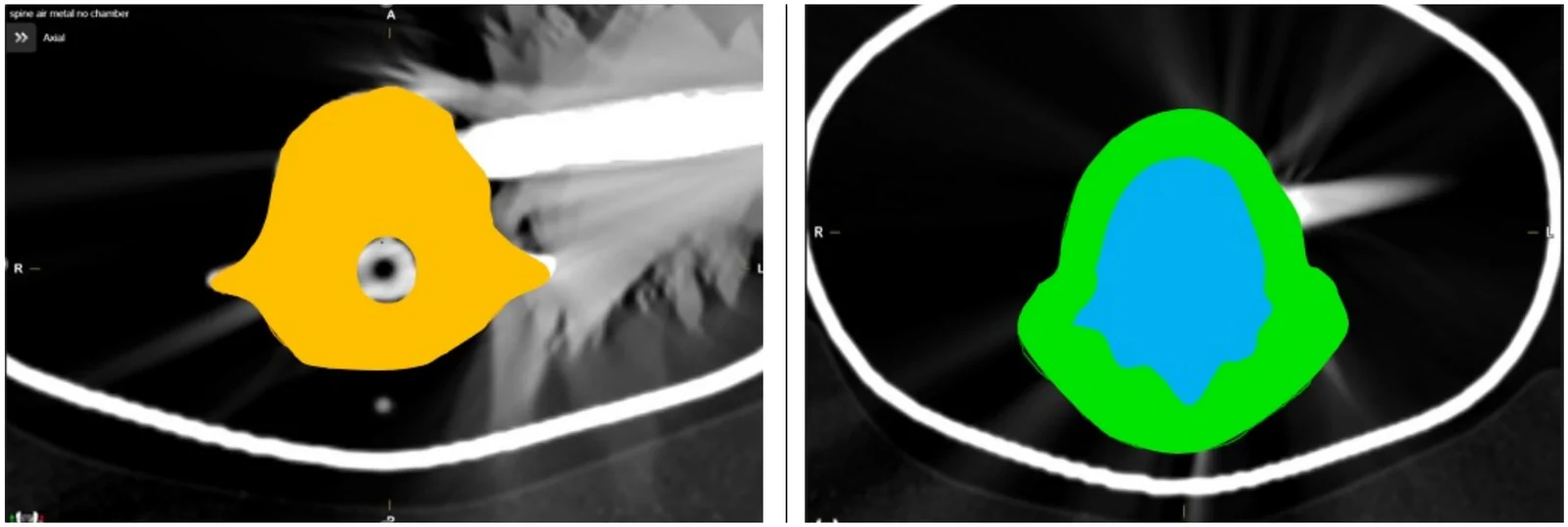
(Left) PTV-spine-metal (orange) is contoured around the vertebrae, and PTV-small-metal (no color) is subtracted from it. Note that the steel rod is inserted into the vertebral spinal A16 insert. (Right) PTV-large-metal (green) and PTV-spine-metal (blue) are displayed in the air-filled spine phantom with a metal insert.

PTV-Spine-Metal was a contour of the vertebrae, which included the metal insert. PTV-Small-Metal was created by expanding the contoured active volume of the ion chamber by 4 mm. PTV-Large-Metal was created by expanding PTV-Spine-Metal by 10 mm. **Figure 3** displays the PTV targets drawn onto the air-filled spine phantom.

Three plans were created using the three PTVs detailed in **Figure 3**. PTV-Expanded was used to create an 8-Gy plan whose target included air, bone, and metal. An avoid plan was created by subtracting PTV-Small-Metal from PTV-Spine-Metal. A prescription of 8 Gy was prescribed to the PTV-Large-Metal structure, and there was a “strict” constraint of D1.6cc <4 Gy and Dmax <5 Gy to the avoid structure (derived off PTV-Small-Metal) with the Guardian slider set to 50. An SIB plan was created by prescribing 8 Gy to PTV-Large-Metal and 10 Gy to PTV-Small-Metal. Furthermore, the artifact generated by computed tomography (CT) scanning the steel rod was contoured and overridden to match the initial ED of the phantom: air = 0.005 g/cc and bone = 1.6 g/cc. Note that these were the material-specific ED values from the original phantom from the air-filled spine phantom plans. The only beam model evaluated for these sets of measurements was 6FFF.

## 3 Results

Beam modeling was performed, and all results were measured in 1 day by a full-time equivalent (FTE) medical physicist and a resident. All MPPG 5.b tests passed their recommended tolerance levels. All test plans passed with a passing rate >90%. The ASG leaf gap results for the MC algorithm were measured to be 0.4 mm for both the 6MV and 6FFF energies, compared to a modeled value of 0.26 mm. The 0.4-mm leaf gap was input into the TPS.

Cranial and water-filled spinal phantoms with an A16 chamber were used to collect data. **Tables 2** and **3** display the results of the 6MV and 6FFF cranial and spinal plans, respectively.

**Table 3:** The TPS calculations using PB v4.0 and v4.5 and MC algorithms were based on the average dose to the active volume of the ion chamber and compared with corrected measurements taken with an A16 ion chamber in a spine phantom filled with water.

Aim 2 spine plans			TPS (Gy)	Measured (Gy)	Difference (%)
Spine large	6MV	PB v4.0	9.01	9.42	-4.55
		PB v4.5	9.18		-2.61
		MC	9.5		0.84
	6FFF	PB v4.0	8.97	9.22	-2.79
		PB v4.5	9.08		-1.54
		MC	9.09		-1.43
Spine avoid	6MV	PB v4.0	3.91	4.56	-16.62
		PB v4.5	4.16		-9.62
		MC	4.56		0.00
	6FFF	PB v4.0	4.12	4.64	-12.62
		PB v4.5	4.32		-7.41
		MC	4.48		-3.57
Spine SIB	6MV	PB v4.0	10.42	10.73	-2.98
		PB v4.5	10.56		-1.61
		MC	10.77		0.37
	6FFF	PB v4.0	10.83	11.12	-2.68
		PB v4.5	10.82		-2.77
		MC	10.84		-2.58

The majority of the MC measurements were within 3% of the expected dose, calculated by the TPS, as quoted by Brainlab and the 5% recommendation of MPPG 9.b (14). There were two MC plans that did not achieve the outlined standard: 6MV Cranial Small and 6MV MultiMet Avoid. Furthermore, there were five PB plans that had an E2E result greater than 5%. Of the five out-of-tolerance PB plans, three of the four avoid plans had E2E results greater than 10% for Elements v4.0, but the measured result was less than 10% when calculating the same plans using the PB v4.5 model. When switching to PB v4.5, the Cranial Small plan went from being out of tolerance with respect to MPPG 9.b standards to passing.

For aim 3, spine phantom measurements were repeated, but water was drained from the phantom to create an air–bone environment and test the system’s ability to operate in a heterogeneous environment. **Table 4** displays the results of the PTV-Large, PTV-Avoid, and PTV-SIB plans.

**Table 4:** An air-filled spine phantom was fitted with an A16 ion chamber. MC and PB v4.0 and v4.5 calculated plans were created and compared with measured values using an A16 ion chamber.

Aim 3 plans			TPS (Gy)	Measured (Gy)	Difference (%)
PTV large	6FFF	PB v4.0	9.39	9.08	3.30
		PB v4.5	9.49		4.32
		MC	9.03		-0.55
PTV avoid	6FFF	PB v4.0	4.39	4.56	-3.87
		PB v4.5	4.58		0.44
		MC	4.62		1.30
SIB	6FFF	PB v4.0	10.06	9.72	3.38
		PB v4.5	10.04		3.19
		MC	9.56		-1.67

Both the MC and PB plans met the 5% E2E margin outlined in MPPG 9.b.

**Table 5** displays the results of the PB and MV TPS calculations compared with the measured values using this setup. All 6FFF measurements matched within 5% threshold used for E2E testing. These results were similar to the performance of the air-filled spine phantom.

**Table 5:** A spine phantom with a steel insert was used to evaluate the TPS MC and PB v4.0 and v4.5 algorithms as well as the beam model.

Aim 4 plans			TPS (Gy)	Measured (Gy)	Difference (%)
Metal large	6FFF	PB v4.0	7.89	7.76	1.65
		PB v4.5	7.88		1.52
		MC	7.72		-0.52
Metal avoid	6FFF	PB v4.0	6.02	5.98	0.66
		PB v4.5	6.02		0.66
		MC	5.9		-1.36
Metal SIB	6FFF	PB v4.0	9.34	9.15	2.03
		PB v4.5	9.41		2.76
		MC	9.12		-0.33

The shifted chamber measurements were not significantly different from the original chamber position when analyzing the results using a paired *t*-test (*p* >> 0.05). The percent error, relative to the prescription dose, was -0.05% ± 0.64. The average absolute dose difference was 0.05 Gy. The maximum percent dose difference came from the Cranial Small plan (1.27%) when shifting the ion chamber volume.

## 4 Discussion

MC is the gold standard algorithm linac-based TPS systems use to calculate dose, and it should be used given the opportunity. All but two MC cranial plans (6FFF Cranial Small 6FFF and 6MV Multimet Avoid) were within the 5% recommended tolerance outlined by MPPG 9.b. The 6FFF Spine avoid plan was slightly greater than the 3% Brainlab quoted. Upon further study of failed Cranial Small plan, we determined that that failure was due to the dose distribution relative to the position of the sensitive volume of the ion chamber. The minimum, mean, and maximum dose of the contoured active volume was 17.60, 22.46, and 25.29 Gy, respectively, which led to high uncertainty when measuring the dose in the phantom. Ideally, the minimum and maximum dose, respectively, are as close as possible to the mean dose of the active volume to ensure a precise link between a measured and a calculated value. Furthermore, the dose to the isocenter, which was located at the center of the active volume, was 24.73 Gy, a -1.1% difference with respect to the measured dose. Typically, it is preferable to contour the active volume of a chamber for an end-to-end measurement analysis because the physical measurement is performed in a chamber with a finite volume. Our approach to contouring the active volume was to focus on the air-filled regions within the chamber volume. However, if there is a high variance in dose distribution within the active volume of the chamber, it is reasonable to report dose to the center of the ion chamber and compare with a measured quantity.

The other-out-of-tolerance MC plans were measured in a region of low dose for a 6MV beam. Typically, relative to the prescribed dose (20 Gy), an expected value of 1.06 Gy would be omitted from the gamma analysis because it is common practice to omit doses less than 10% of the prescribed dose (14). As such, an absolute dose difference of 0.16 Gy in the low-dose region of the treatment plan is not clinically consequential despite the large percent difference between calculated and measured doses. Similarly, the MC-calculated Spine Avoid plan has a percent difference of -5.94% between the TPS and the measured value based on a 0.26-Gy difference.

The uncertainty budget for this study could be traced from the TPS and physical measurements: MC uncertainty (TPS), cross-calibration uncertainty (physical), and positional uncertainty (physical). The MC parameters defined in the TPS for this study and all clinical treatments were 2% dose calculation using a 1-mm dose grid. Furthermore, cross-calibration was performed by measuring the absorbed dose to water using a NIST-traceable calibrated PTW Semiflex ionization chamber and creating a calibration coefficient for the A16 ionization chamber to equal the measured absorbed dose of the Semiflex. Per TG-51a, it is reasonable to calculate about 1% uncertainty from sources such as beam quality, polarity and recombination effects, N_D,w_, and k_Q_ (15). We assumed that all of these factors account for uncertainty for both the Semiflex and the A16 calibrations. It is worth noting that the reference field charge readings were stable for both the Semiflex and A16. Positional uncertainty was another consideration for this study. Small fields are impacted more significantly by penumbral effects because of their size relative to the width of the penumbra. Based on the relative uncertainty analysis that we performed, we could reasonably surmise that a majority of our measurements fall within one standard deviation (0.64%) of the prescribed dose. The approximately 1% of the cross-calibration and 0.64% of the positioning physical uncertainty budget provide reasonable uncertainty estimate and closely matches the quoted uncertainty of the MC calculation algorithm.

All plans were generated in the same manner to ensure consistency. The plans did not push the optimizer to create the “best” possible plan by trying to reduce the conformity and gradient indices. The sliders were set to the neutral position in the Elements planning software. However, the purpose of this paper was not determining how to generate the best possible plan; it was to evaluate the accuracy and efficacy of an accelerated beam model and its associated TPS algorithm in a clinically relevant environment. Cranial and spine plans were created in their respective anthropomorphic phantoms. Extreme planning volume densities tested the TPS algorithms by creating a variety of air–bone and air–bone–steel plans in the TPS.

Regarding aims 3 and 4, only the 6FFF beam energies were used to evaluate spine SRS studies. The 6FFF beam energy is forward-peaked, which provides some conformity advantages, and using these FFF beams is common practice when planning such cases. Since multimet cranial treatment planning can include off-axis targets, there was a perceived possibility of benefits for a 6MV beam. For this reason, this model was also created and validated. Clinically, we have not found real benefits of 6MV and only utilize 6FFF for clinical treatment plans. Furthermore, the 6MV beam energy was used to evaluate spine SRS planning in a clinically relevant environment, a water-filled spine phantom.

When the phantom was scanned with the steel insert, we had to manage the artifacts generated. ED was copied from the prior air-filled spine phantom image set, and the artifacts were contoured and overridden to replicate the prior data. There is no reason to believe that this technique created spurious results during planning or the measurement phase. Furthermore, Brainlab Elements does offer a preset list of ED for a variety of metal implants within the Tissue Modeling application. However, if the ED quoted by the manufacturer or a measured ED is not available, it is possible to manually enter an ED.

Furthermore, three out of four PTV avoid plans calculated with PB v4.0 had a difference of over 10% with respect to measured values, and both small field cranial plans failed the 5% threshold previously established. The avoid plans for the PBv4.5 model agree within 10%, and the 6FFF small field cranial plan exceeds the 5% threshold (while the 6MV plan passes). The high error plans (>10%) can be attributed to poor modeling of scatter when using the PB algorithm. A possible explanation could be that the water-filled spine phantom will induce more scattering events with respect to an air-filled environment, hence a greater compounding of calculation error in the water-filled phantom. Furthermore, the small field cranial plans did not perform to clinical standards. Small field dose calculation should be performed in MC as well due to the significant dosimetric impact a lack of lateral charged particle equilibrium has on small field sizes. Note that, per the vendor, the PB v4.5 used the same calculation algorithm; however, the PB dose profiles were re-created and re-fitted with optimized parameters to improve the results for Cranial and Spine SRS planning.

MPPG 5.b testing is well documented and established and does not need to be emphasized as part of this work. However, emphasis should be placed on patient-specific QA plan testing as part of the commissioning process for an SRS/SRT program due to dosimetry impacts of MLC modeling on small fields. The benefit of patient-specific QA testing stems from being able to see the entire dose distribution of a plan instead of a single point, thus allowing for an evaluation of all machine parameters, including MLC/jaw penumbra and small field OF uncertainties, integrated into a plan delivery. Furthermore, a clinical database spanning ~3 years SRS and SRT plans, typically utilizing high modulation, for both single and multiple lesions passing patient-specific QA demonstrates accurate beam modeling.

It is worth noting that during the tissue modeling section of this study, it is possible for the Elements software to omit portions of air within an external body. These sections will not have an ED assigned to them and will therefore not be considered during the dose calculation phase. As such, it is important to review the external (or outer contour) for any holes where air is present to ensure that dose is properly calculated throughout the volume. This occurrence near the PTV and/or GTV will cause serious errors in dose calculation. A simple technique to verify that this problem does not occur within the target volumes is to ensure that the minimum PTV dose is not at or near 0 Gy. This phenomenon is prone to occur in the auditory canal, sinus cavity, and an air-filled phantom.

## Data Availability

The original contributions presented in the study are included in the article/supplementary material. Further inquiries can be directed to the corresponding author.
